# Plasma Level of Soluble ST2 in Chronically Infected HIV-1 Patients with Suppressed Viremia

**DOI:** 10.2174/1874613601711010032

**Published:** 2017-04-26

**Authors:** Mehwish Younas, Christina Psomas, Vikram Mehraj, Renaud Cezar, Pierre Portales, Edouard Tuaillon, Adeline Guigues, Jacques Reynes, Pierre Corbeau, Jean-Pierre Routy

**Affiliations:** 1 Institute for Human Genetics, CNRS UPR1142, 141 rue de la Cardonille, 34396 Montpellier cedex 5, France,; 2 Infectious Diseases Department, University Hospital, 80 avenue A. Fliche, 34295 Montpellier cedex 5, France,; 3 Research Institute and Chronic Viral Illness Service of the McGill University Health Center, Montréal, Québec, Canada; 4 Immunology Department, University Hospital, Place du Pr Debré, 30029 Nîmes cedex, France,; 5 Immunology Department, University Hospital, 80 avenue A. Fliche, 34295 Montpellier cedex 5, France,; 6 Microbiology Department, University Hospital, 371 Av. du Doyen Gaston Giraud, 34295 Montpellier cedex 5, France,; 7 UMI 233, IRD-Montpellier I University, 911 avenue Agropolis, 34294 Montpellier cedex 5, France,; 8 Montpellier I University, 5 Boulevard Henri IV, 34967 Montpellier cedex 2, France,; 9 Division of Hematology, McGill University Health Center, Montréal, Québec, Canada

**Keywords:** Interleukin-33, Plasma, HIV, Viremia, Cardiovascular, Translocation

## Abstract

**Introduction:**

Interleukin-33 (IL-33) is a cell damage-induced alarmin. The plasma concentration of suppression of tumorogenicity (sST2), a surrogate marker of IL-33 production, is a prognostic marker of cardiovascular disease.

**Observation:**

Recently, we reported that sST2 plasma levels were elevated in early HIV-1 infection and linked to markers of microbial translocation and of T cell activation.

**Results:**

Here we show that it is not the case in patients with suppressed viremia. Thus, IL-33 plays its alarmin role only during the early phase of the infection.

## INTRODUCTION

Interleukin-33 (IL-33), which is a member of the interleukin-1 (IL-1) cytokine family, is produced in response to the cell damage caused by infections or by breaches in tissue barrier integrity [1]. The soluble form of the IL-33 cognate receptor, suppression of tumorogenicity (sST2), acts as a decoy receptor; the plasma concentration of sST2 is a surrogate marker of IL-33 production and a prognostic marker of sepsis, acute respiratory distress, and of heart failure [1]. Recently, we reported that sST2 plasma levels were elevated in early HIV-1 infection [2]. Furthermore, sST2 levels correlated with the levels of intestinal-type fatty acid-binding protein (I-FABP), a marker of epithelial gut damage. We also found a correlation between plasma sST2 concentrations and the following markers of immune activation: CD8+ T-cell count, percentage of HLA-DR+CD38+CD4+ T-cells, percentage of HLA-DR+CD38+CD8+ T-cells, percentage of PD-1 CD4+ T-cells, soluble CD14 (sCD14), soluble CD40L, interferon-γ, and plasma indoleamine-2,3-dioxygenase activity. In the recent ACTIVIH study, we analyzed 68 markers of residual immune activation in 120 aviremic, HIV-1-infected adults treated with antiretroviral therapy [3]. We decided to measure this cohort’s plasma sST2 concentrations.

## METHODOLOGY

sST2 concentrations were determined by ELISA (Quantikine ELISA kit, R&D Systems) in stored, frozen plasma. sST2 concentrations were compared using unpaired two-tailed t test.

### Findings

**Fig. (1):**
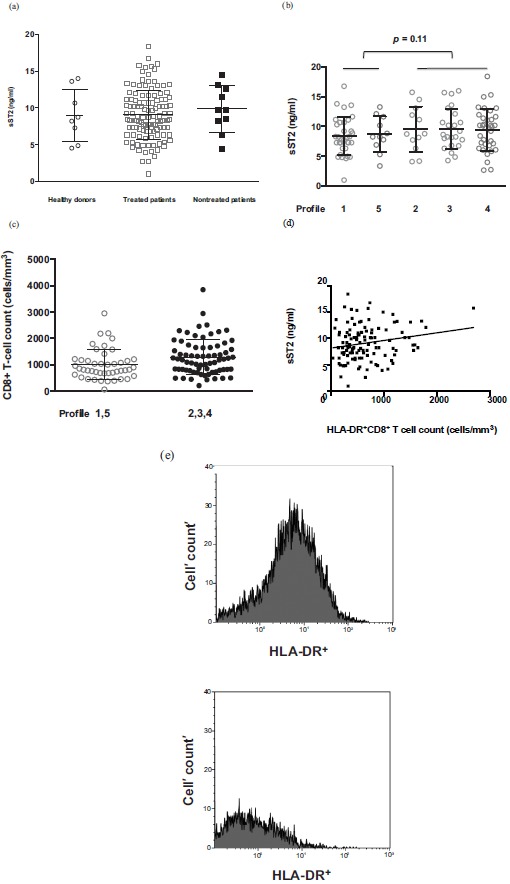
**Plasma sST2 concentrations during chronic HIV-1 infection.** (a) Plasma sST2 concentrations in uninfected controls, and in treated or untreated, chronically infected adults. (b) Plasma sST2 concentrations in chronically infected adults with various immune activation profiles. (c) CD8+ T-cell count of patients presenting with various immune activation profiles. (d) Correlation between plasma sST2 concentration and the number of circulating CD8+ T-cells expressing CD38. (e) HLA-DR expression on CD8+ T cells from patients with sST2 levels of 15.7 (upper plot) and 2.7 (lower plot) ng/mL.

The 120 virologic responders we analyzed were mostly male (82%) and Caucasians (95%). Their duration of viral suppression (mean ± SD) was 102 ± 47 months, for a duration of infection of 17.2 ± 7.4 years. Their current and pretherapeutic CD4 counts were 688 ± 326 and 192 ± 108 cells/μL, respectively. Almost all of them were infected by cytomegalovirus (91%) and Epstein-Barr virus (98%), and only 5% by Hepatitis C virus. Consistent with our previous findings [2] and with those of Secemsky et al. [4], we found no difference in plasma sST2 concentrations between effectively-treated HIV-1-infected adults and age- and sex-matched healthy controls (Fig. **1a**). Although the concentration of sST2 was higher in untreated patients, this difference was not statistically significant (10.7 ± 1.0 and 9.7 ± 0.3 ng/mL, respectively, *p* = 0.42). sST2 plasma levels were not linked to the duration of viral suppression (Pearson r < 0.01, p = 0.93).

We subsequently looked for a link between plasma sST2 concentration and markers of causes of immune activation. Residual viremia, coinfections, microbial translocation, immune senescence and Treg deficiency have been identified as potential drivers of persistent immune activation in virologic responders [5]. We failed to find a correlation between plasma sST2 levels and i) residual viremia below 50 copies/mL or with frequency; ii) markers of senescence on CD4+ or CD8+ T-cells (CD27, CD28 and CD57) or on NK cells (CD57); iii) cytomegalovirus, Epstein-Barr virus, hepatitis A virus, hepatitis B virus, and/or hepatitis C virus coinfections; iv) the plasma concentrations of bacterial DNA, I-FABP or lipopolysaccharide-binding protein or v) the percentage of Treg cells. Furthermore, there was no correlation between plasma sST2 concentration and smoking tobacco.

We also looked for correlations between plasma sST2 levels and the phenotype of the immune activation observed in these patients. In the ACTIVIH study, a double hierarchical clustering of the 68 markers of immune activation and of the 120 patients resulted in the identification of 5 patient groups presenting with very different immune activation profiles [3]. This means that patients belonging to the same profile have common marks of immune activation as determined by the 68 markers we used. Looking for differences in sST2 levels among these immune activation profiles, we observed that patients with profiles 2, 3, and 4 tended to have higher sST2 concentrations than the group consisting of participants with profiles 1 or 5 (9.5 ± 0.4 and 8.5 ± 0.5 ng/mL, respectively, *p* = 0.11; Fig. **1b**). Of note, immune activation profile 2, which we previously showed to be strongly linked to an atherothrombosis-associated metabolic syndrome [3], correlated with a high sST2 concentration, which is prognostic of cardiovascular disease [6]. In line with our previous observation of a link between sST2 concentration and CD8+ T-cell count in early HIV-1 infection [2], we found here that chronically infected patients with immune activation profiles 2, 3 and 4 presented with higher CD8+ T-cell counts than patients with immune activation profiles 1 and 5 (*p* = 0.02; Fig. **1c**).

We also looked for correlations between plasma sST2 levels and each of the 68 activation markers. No correlation was observed between plasma sST2 levels and i) CD4+ or CD8+ T-cell differentiation (naïve/central memory/effector memory), CD4+ T-cell activation (HLA-DR and/or CD38), CD4+ or CD8+ T-cell exhaustion (PD-1); ii) NK cell activation (HLA-DR and/or CD69) or dysfunction (loss of CD56 expression); iii) B cell activation (IgG, IgA, and IgM plasma levels); iv) monocyte activation (sCD14, sCD163); v) neutrophil activation (CD64, PD-L1, loss of CD62L expression); vi) inflammation (CRP, soluble TNF receptor I); vii) endothelial cell activation (tissue plasminogen activator, soluble endothelial protein C receptor and thrombomodulin); and viii) fibrinolysis (D-dimer). However, we found a link between sST2 levels and the number of CD8+ T-cells expressing the activation marker HLA-DR (Pearson r = 0.19, *p* = 0.03 ; Fig. **1d**). This is illustrated in Fig. (**1e**) by the difference in HLA-DR+CD8+ T cell count in two patients presenting with sST2 plasma levels of 15.7 and 1.0 ng/mL, respectively.

## CONCLUSION

Collectively, our data showed that sST2 is mostly increased during early infection. HIV-induced gut barrier damage is major at this stage, being partly reduced under antiretroviral therapy [7]. IL-33 is produced by damaged endothelial and epithelial cells at this barrier site [1]. Accordingly, we observed a high sST2 peripheral blood concentration, linked to the levels of the microbial translocation markers I-FABP and sCD14 in early HIV infection [2]. It is thus logical that circulating sST2 levels are higher during the initial phase than during the treated chronic phase of the infection. IL-33 signaling via ST2 inhibits the development of atherosclerosis, and therefore, sST2 is thought to be proatherogenic [8]. Consequently, a slight sST2 overproduction in treated HIV patients, over years, might favour atherothrombosis. This could explain why sST2 level is a predictor of cardiovascular insufficiency in HIV patients aviremic under treatment [4] as it is in non-infected adults [6].
